# The effect of β-alanine and NaHCO_3_ co-ingestion on buffering capacity and exercise performance with high-intensity exercise in healthy males

**DOI:** 10.1007/s00421-014-2895-9

**Published:** 2014-05-16

**Authors:** Jessica Danaher, Tracey Gerber, R. Mark Wellard, Christos G. Stathis

**Affiliations:** 1 College of Health and Biomedicine, Victoria University, Melbourne, Australia; 2 Queensland University of Technology, Brisbane, Australia; 3 Institute of Sport, Exercise and Active Living and the College of Health and Biomedicine, Victoria University, Melbourne, VIC 8001 Australia

**Keywords:** Beta-alanine, Carnosine, Bicarbonate, High-intensity exercise, Metabolism

## Abstract

**Introduction:**

β-alanine (BAl) and NaHCO_3_ (SB) ingestion may provide performance benefits by enhancing concentrations of their respective physiochemical buffer counterparts, muscle carnosine and blood bicarbonate, counteracting acidosis during intense exercise. This study examined the effect of BAl and SB co-supplementation as an ergogenic strategy during high-intensity exercise.

**Methods:**

Eight healthy males ingested either BAl (4.8 g day^−1^ for 4 weeks, increased to 6.4 g day^−1^ for 2 weeks) or placebo (Pl) (CaCO_3_) for 6 weeks, in a crossover design (6-week washout between supplements). After each chronic supplementation period participants performed two trials, each consisting of two intense exercise tests performed over consecutive days. Trials were separated by 1 week and consisted of a repeated sprint ability (RSA) test and cycling capacity test at 110 % Wmax (CCT_110 %_). Placebo (Pl) or SB (300 mg kgbw^−1^) was ingested prior to exercise in a crossover design to creating four supplement conditions (BAl-Pl, BAl-SB, Pl–Pl, Pl-SB).

**Results:**

Carnosine increased in the gastrocnemius (*n* = 5) (*p* = 0.03) and soleus (*n* = 5) (*p* = 0.02) following BAl supplementation, and Pl-SB and BAl-SB ingestion elevated blood HCO_3_^−^ concentrations (*p* < 0.01). Although buffering capacity was elevated following both BAl and SB ingestion, performance improvement was only observed with BAl-Pl and BAl-SB increasing time to exhaustion of the CCT_110 %_ test 14 and 16 %, respectively, compared to Pl–Pl (*p* < 0.01).

**Conclusion:**

Supplementation of BAl and SB elevated buffering potential by increasing muscle carnosine and blood bicarbonate levels, respectively. BAl ingestion improved performance during the CCT_110 %_, with no aggregating effect of SB supplementation (*p* > 0.05). Performance was not different between treatments during the RSA test.

## Introduction

High-intensity exercise (HIE) results in the accumulation of glycolytic metabolites as a consequence of anaerobic metabolism during times of limited oxygen availability to the working cell (Coso et al. 2010; Sweeney et al. 2010). Although not the primary cause, an increased acidity of the working cells caused by hydrogen ions (H^+^) accumulation as by-products of anaerobic metabolism has potentially deleterious effects implicated in fatigue (Begum et al. 2005; Bishop et al. 2004) and can lead to significant impairments to exercise performance at high intensities (Robergs et al. 2005). Potential limiting factors resulting from acidosis include direct influences on contraction within the muscle cell due to competition of the H^+^ with Calcium ion (Ca^2+^) (Fabiato and Fabiato 1978), inhibition of glycolytic enzymes (Sutton et al. 1981) and PCr recovery (Harris et al. 1976), and its interference to the buffering process (Sahlin and Harris 2011).

Intramuscular pH homeostasis is achieved by active and passive transport of H^+^ out of the muscle cell into the surrounding interstitium by physiochemical buffers, which work to maintain pH within a range for optimal enzyme function by tempering the effect of acidosis in the intracellular and extracellular milieu during HIE (Hill et al. 2007; Siegler et al. 2010). Physiochemical buffers include muscle carnosine, which plays an immediate role due to its intracellular location, and blood bicarbonate (HCO_3_^−^), which has an extracellular presence (Sale et al. 2010).

Carnosine (β-alanyl-l-histidine) is found in relatively high concentrations in human skeletal muscles at around 5–10 mmol kg^−1^ wet weight (ww) (Boldyrev and Severin 1990; Harris et al. 2006; Hill et al. 2007). It is endogenously synthesised from β-alanine (the limiting precursor in its synthesis) and l-histidine, and contributes between 8 and 15 % of the endogenous H^+^ buffering capacity (Van Thienen et al. 2009; Baguet et al. 2010). Its synthesis is slow and can take several weeks to accumulate in the muscle, warranting the need for chronic supplementation of β-alanine (Baguet et al. 2009; Derave et al. 2007). Increases in intense exercise performance following β-alanine ingestion indicate the potential of its influence on the intracellular pH buffer capacity via elevated carnosine concentrations (Hill et al. 2007; Sale et al. 2010; Sweeney et al. 2010). Although specific cellular mechanism(s) of the action and the consequential benefit to exercise performance remains unclear, elevated carnosine has also been linked to various additional cellular functions including, the regulation of Ca^2+^ sensitivity (Boldyrev and Severin 1990; Johnson and Aldstaft 1984), the protection of protein glycosylation by acting as a sacrificial peptide (Hipkiss et al. 1995), the inhibition of protein cross-linking by reacting with protein–carbonyl groups (Hipkiss 2000), and free radical scavenging by acting as an antioxidant (Chasovnikova et al. 1990).

Blood HCO_3_^−^ is important for maintaining the homeostatic acid-base balance in the body (McNaughton et al. 2008). It also plays a role in attenuating the influence of the acute acid load following intense exercise once intracellular buffering capacity is exceeded and H^+^ diffuses into the bloodstream (Juel et al. 2004; Swank and Robertson 1989), and may inevitably assist in postponing the potential effects of fatigue (McNaughton et al. 2008; Swank and Robertson 1989). Thus, the concentration of HCO_3_^−^ present in the blood prior to exercise potentially influences both extracellular buffering capacity and the extent to which the H^+^ is able to diffuse out of the active muscle (Price et al. 2003). NaHCO_3_ ingestion has the potential to influence skeletal muscle function and prolong performance due to an improvement in blood buffering potential via the creation of an enhanced electro-chemical pH gradient between the intracellular and extracellular milieu, consequently allowing for a greater efflux of H^+^ out of the muscle cell (Bishop et al. 2004; Price et al. 2003).

Combined nutritional strategies which enhance muscle carnosine and blood HCO_3_^−^ content, such as oral supplementation of β-alanine and NaHCO_3_, respectively, are therefore potentially ergogenic, particularly during HIE protocols with high glycolytic contribution, where acidosis may be a limiting factor to exercise performance (Price and Simons 2010; Sale et al. 2011). Although many previous studies have investigated the use of either β-alanine or NaHCO_3_ independently in improving parameters associated with HIE, little is known regarding the ergogenic effects of their co-ingestion.

Whilst recently published reports have examined the effects of this ergogenic strategy on continuous bout HIE (Bellinger et al. 2012; Sale et al. 2011), no study to date has, to the authors knowledge, investigated its effects on intermittent HIE or implemented a crossover design to account for individual variability. The aim of this study was therefore to investigate the effect of the co-consumption of β-alanine and NaHCO_3_ on HIE performance in healthy males. It was hypothesised that manipulating intra- and extracellular buffering capacity, via the co-ingestion of β-alanine and NaHCO_3_, respectively, will improve HIE performance variables compared to the placebo condition or the ingestion of either supplement alone. The co-consumption of β-alanine and NaHCO_3_ will buffer acidic by-products generated from anaerobic glycolysis, and thus enhance the ability to perform during both an intense intermittent and continuous exercise bout.

## Methods

### Participants

Eight apparently healthy, recreationally active males (26.2 ± 1.9 year; 79.8 ± 2.11 kg; 179.0 ± 2.2 cm; VO_2peak_ 51.0 ± 2.5 ml kg^−1^ min^−1^) volunteered to take part in this study, which was approved by the Victoria University Human Research Ethics Committee (HRETH 11/11) and performed in accordance with the ethical standards set out in the 1964 Declaration of Helsinki. After completing a medical questionnaire, each participant signed informed consent forms and presented for preliminary testing.

Participants were asked to refrain from consuming caffeine and alcohol, and from undertaking strenuous exercise 24 h prior to all experimental trials. Participants recorded their dietary intake 24 h before the first day of the first experimental trial, and were asked to consume a similar dietary intake the day prior to all subsequent trials. Experimental trials were conducted in the morning, approximately 10–12 h after the last meal. A standardised warm-up period of 5 min of cycling at 80 W was performed on an Excalibur Lode Cycle ergometer (Netherlands) before each trial, followed by 5 min rest. All tests were followed by 60 min passive recovery in a supine position.

### Preliminary testing

Peak oxygen consumption (VO_2peak_) was determined approximately 1 week before the beginning of each chronic supplementation period. A standard graded exercise protocol on a Excalibur Lode Cycle ergometer (Netherlands) of 3 × 3 min sub-maximal workloads at 50, 100 and 150 W followed by successive 1-min workload increments of 25 W until volitional exhaustion. Participants were encouraged to maintain a pedal frequency between 80 and 100 RPM and the test was terminated when RPM could not be maintained >80 RPM for a period of 5 s. Expired air was directed by a Hans Rudolph valve via a ventilometer (Moxus; AEI Technologies, Pennsylvania, USA) into a mixing chamber and analysed for oxygen and carbon dioxide content. Prior to each VO_2peak_ test, the gas analyser was calibrated using commercially prepared gas mixtures (BOC Gases, Australia). Following preliminary testing, participants were familiarised with the exercise protocols in which they would be participating in during the experimental trials.

### In vivo magnetic resonance spectroscopy (MRS) analysis

MRS was used in this study as a non-invasive quantification of human muscle carnosine before and after the β-alanine and placebo chronic supplementation periods in *n* = 5 participants (due to access and availability of MRS). These measurements were made on a whole body proton 3 tesla scanner (Siemens Trio Tim, Germany) equipped with a flexible knee coil at The Royal Children’s Hospital (Melbourne, Australia). Muscle carnosine concentration was quantified in the gastrocnemius and soleus muscles of the right leg throughout the study using a voxel of dimension 40 mm × 30 mm × 12 mm. Data were acquired using a standard PRESS sequence with an echo time of 30 ms, a relaxation delay of 2,000 ms and 176 transients. Muscle carnosine concentration was determined by comparing data recorded from the gastrocnemius and soleus muscles to a standard curve generated from external carnosine reference phantoms for absolute quantification. External carnosine reference phantoms contained 6.32, 30.00 and 58.93 mM carnosine (Sigma) with sodium azide (NaN_3_) as an antibacterial agent. No correction was made for differences in relaxation time.

A line broadening of 1 Hz was applied to the data prior to Fourier transformation to reduce any noise which may have altered the accuracy of results. Manual phasing was then applied before line fitting the spectra of each scan with an AMARES algorithm (jMRUI, version 4.0, build 113) (Naressi et al. 2001; Vanhamme et al. 1997). Peak amplitude (arbitrary scale) and linewidth (lw) results attained through this algorithm were then used to determine the signal intensity (area of each peak) using a Lorentzian equation; (I, Intensity; w, 0.5 × lw at 1/2 maximum height; 2*θ*_0_, position of peak max):

$$I(2\theta ) \, = \, w^{2} / \, w^{2} + { (}2\theta \, - \, 2\theta_{0} )^{2} .$$

Each spectrum contained two peaks, corresponding to the C_2_–H and C_4_–H imidazole protons of carnosine, and therefore the signal intensity of each peak was combined after adjusting to account for the number of protons in the structures giving rise to the peaks. A standard curve was then generated based on the concentration of each phantom scan and the signal intensity (area calculated from the phantom peak). The apparent concentration of carnosine from the gastrocnemius and soleus muscle scans was then determined with reference to the standard curve.

### Supplementation protocols

**Fig. 1:**
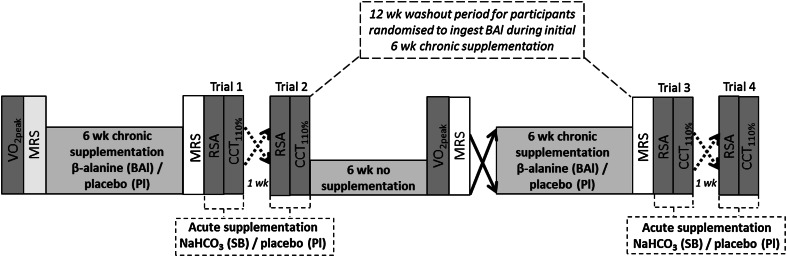
Design of the study. Each trial consisted of two exercise tests performed over consecutive days. A total of 12 weeks between trials 2 and 3 was implemented to ensure adequate supplement washout time participants randomised to ingest β-alanine during the initial chronic supplementation. *MRS* Magnetic resonance spectroscopy, *RSA* repeated sprint ability test, *CCT*_*110* *%*_ cycling capacity test. *Solid arrows* depict crossover between acute supplementation (Pl and SB). *Dotted arrows* depict crossover between chronic supplementation (BAl and Pl)

The dual supplementation, double-blind, crossover experimental design was randomised for both β-alanine vs. placebo and NaHCO_3_ vs. placebo (Fig. 1) and all participants received the four supplement combinations. Specifically, there were two periods of 6-week chronic supplementation of capsulated β-alanine (Musashi, Australia) or the placebo calcium carbonate (CaCO_3_) (E170, Melbourne Food Ingredient Depot, Australia) which were separated by a minimum washout period of 6 weeks (no supplementation). The β-alanine supplementation procedure consisted of 4.8 g day^−1^ (6 × 800 mg) for 4 weeks and then 6.4 g day^−1^ (8 × 800 mg) for 2 weeks. CaCO_3_ was administered in equal mass amounts as β-alanine. Participants were asked to spread their consumption of daily doses of β-alanine/CaCO_3_ evenly throughout the day to avoid any possible paraesthesia. To facilitate compliance, a supplement diary was provided to participants upon commencing each chronic supplementation bout.

To investigate the superimposition of NaHCO_3_ with β-alanine, the acute administration of NaHCO_3_ (Sodibic, Aspen Pharmacare, Australia) occurred following each of the 6-week periods of β-alanine and placebo supplementation. This required two trials of either 300 mg kgbw^−1^ NaHCO_3_ or placebo (CaCO_3_) supplementation in a randomised crossover design. This acute supplement ingestion was administered 90 min prior to the exercise bouts of the respective trials and was split into 6 equal doses over the first 50 min of the 90-min pre-exercise period. Participants were asked to consume 300 ml of water with each dose to assist in avoiding potential gastrointestinal side effects. These trials were separated by 7 days, performed in a randomised order, and β-alanine or respective placebo supplementation was extended until the second acute supplementation protocol was completed. Ultimately, the following four supplementation combinations were generated: β-alanine + CaCO_3_ (BAl-Pl), β-alanine + NaHCO_3_ (BAl-SB), CaCO_3_ + CaCO_3_ (Pl–Pl), and CaCO_3_ + NaHCO_3_ (Pl-SB).

### Experimental trial protocols

Participants were asked to complete 2 exercise tests, over consecutive days, at the end of each of the aforementioned four co-supplement periods (Fig. 1). These tests were designed to produce high acid loads whilst allowing examining the effect of supplementation on both intermittent and continuous HIE. The first test, a repeated sprint ability test (RSA), consisted of 5 repeats of 6 s maximal effort cycling bouts separated by 24 s rest (1:4 work-to-rest ratio). Peak and average power output were recorded after each sprint. This test was performed using a Wattbike Pro (British Cycling, Nottingham) and the same level of resistance was set for all trials (Air Brake 7.0). All sprints were performed in the standing position beginning with level pedals and the right leg forward, and with strong verbal encouragement. During the 24 s recovery intervals between sprints, participants were instructed to rest passively.

The second exercise test was a cycling capacity test (CCT_110 %_) performed at 110 % of the workload achieved at VO_2peak_ prior to each chronic supplementation period. Participants were required to cycle in a seated position at 110 % of their peak power output (*W*_max_) (335.9 ± 16.6 W) between 80 and 100 rpm for as long as possible. The clock was stopped at the point where their cadence could not be sustained above 80 rpm (time to exhaustion; TTE). To overcome the inertia of the electromagnetic resistance of initial exercise at low cadence, the protocol on the electrically loaded bike the beginning of the CCT_110 %_ was modified to begin with 15 s of 80 % *W*_max_ and 15 s of 90 % *W*_max_ before 110 % *W*_max_ was attained.

### Blood sampling, treatment and analysis

Blood was sampled from a vein in the antecubital space and the cannula was kept patent with isotonic saline (0.9 % NaCl, Pfizer). Blood was sampled at rest (pre- and post-NaHCO_3_/placebo supplementation), immediately after exercise and during a 60 min post exercise recovery period. One portion of the blood was immediately placed into lithium heparin (BD Vacutainer) tubes and centrifuged at 12,000 rpm for 2 min. Plasma was decanted and stored at −80 °C for later analysis of lactate concentration (YSI 2300 STAT; Yellow Springs Instruments, Ohio, USA). Blood pH and HCO_3_^−^ were analysed immediately using a Radiometer ABL800 FLEX Blood Gas Analyser (ABL800 FLEX; Radiometer Medical, Brønshøj, Denmark).

### Statistical analysis

Results are expressed as mean ± standard error of the mean (SEM). Two-way analysis of variance (ANOVA) with repeated measures was performed (GraphPad Prism 6, Windows XP) one within group factor (time or sprint) and one between group factor (treatment), for blood, plasma and RSA data. Where there was an interaction between the factors, Tukey’s post hoc analysis was performed. One-way AVOVA with repeated measures was performed for CCT_110 %_ and muscle carnosine data (individual × treatment), with paired two-tailed *t* tests when interactions between factors were found. The level of probability required to reject the null hypothesis was set at *p* < 0.05.

## Results

### Muscle carnosine content

**Fig. 2:**
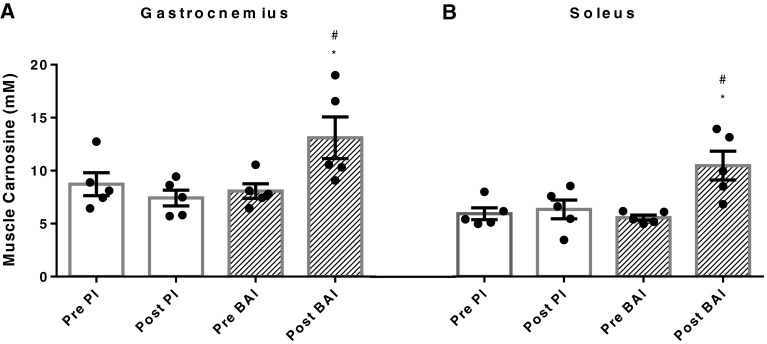
Changes in carnosine concentration in the gastrocnemius (**a**) and soleus (**b**) pre- and post-6-week chronic supplementation of β-alanine (BAl) and placebo (Pl) for *n* = 5. Values expressed as mean ± SEM. *p < 0.05 from Pl at 6 weeks (post), ^#^ *p* < 0.05 from pre-supplementation

The carnosine concentration was elevated in the gastrocnemius and soleus muscles following 6 weeks of supplementation of β-alanine (Fig. 2). In the gastrocnemius muscle (Fig. 2a), the absolute carnosine was 8.08 ± 0.68 and 8.73 ± 1.08 mM in the β-alanine and placebo groups, before supplementation, respectively, and increased by 5.03 ± 1.44 mM (+62 %, to 13.11 ± 1.97 mM; *p* = 0.03) after supplementation, whereas it remained stable at 7.42 ± 0.74 mM (*p* = 0.20) in the placebo group. Six weeks of chronic supplementation also caused the carnosine content of the soleus muscle (Fig. 2b) to increase by 4.92 ± 1.28 mM (+88 %, 5.57 ± 0.25 to 10.48 ± 1.35 mM; *p* = 0.02) before and after supplementation, respectively, in the β-alanine group whilst the carnosine concentration of the placebo group did not change (+7 %, 5.94 ± 0.56 to 6.33 ± 0.89 mM; *p* = 0.6). Carnosine concentrations in both muscle groups were also significantly elevated compared to that of the placebo (Pl) condition following 6 weeks of supplementation [gastrocnemius, +5.69 ± 2.08 (*p* = 0.05); soleus +4.15 ± 0.78 (*p* = 0.006)].

**Fig. 3:**
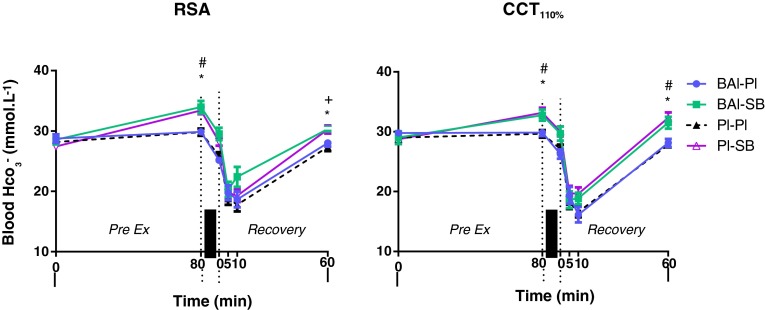
Blood HCO_3_^−^ (mmol L^−1^) changes for study groups during the repeated sprint ability (RSA) and cycling capacity test (CCT_110 %_). Values expressed as mean ± SEM. *p < 0.05 BAl-SB and Pl-SB from Pl–Pl, ^#^ *p* < 0.05 BAl-SB and Pl-SB from PreEx0, ^+^ *p* < 0.05 Pl-SB from PreEx0, *thick vertical bar* denotes exercise

The washout protocol of carnosine from the muscle for participants who received β-alanine during the first chronic supplementation period (*n* = 3) was modified at the end of the experimental procedures, once the supplementation order randomisation was de-coded. This was done to ensure a minimum 12-week washout period upon cessation of β-alanine supplementation, as estimated from previous research (Baguet et al. 2009; Derave et al. 2007). In these participants, the post-supplementation sample in the second of the 6-week supplementation periods (placebo) was unmodified; however, values observed via MRS pre-β-alanine supplementation (initial MRS) were used for baseline data in these participants when examining the effect of chronic placebo supplementation. This was due to the second chronic supplementation period beginning during the 6 week of the washout period, and hence data collected here did not accurately reflected basal muscle carnosine concentrations. After a washout of 6 weeks, carnosine concentrations had decreased to 10.03 ± 1.34 mM (−19 %) in the gastrocnemius and 6.54 ± 0.60 mM (−23 %) in the soleus, at a rate of −0.3 mM week^−1^ and −0.4 mM week^−1^, respectively. Twelve weeks following cessation of β-alanine supplementation (end of second chronic supplementation period), muscle carnosine concentrations in both the gastrocnemius and soleus had returned to baseline concentrations, with the average rate of carnosine consistently decreasing in the gastrocnemius by 0.5 ± 0.03 mM week^−1^ and in the soleus by 0.4 ± 0.02 mM week^−1^. This information demonstrates that a washout period of 12 weeks was adequate to employ a randomised crossover experimental design, with an overall average rate of carnosine concentration decrease following the cessation of β-alanine supplementation.

### Blood HCO_3_^−^ concentration and pH

**Fig. 4:**
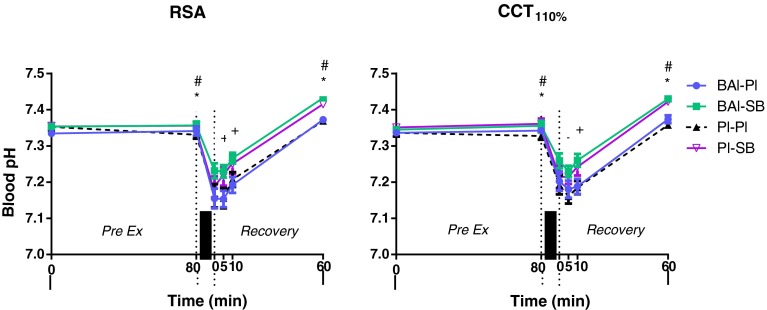
Blood pH recorded for the different groups during the repeated sprint ability (RSA) and cycling capacity test (CCT_110 %_). Values expressed as mean ± SEM. *p < 0.05 BAl-SB and Pl-SB from Pl–Pl, ^#^ *p* < 0.05 BAl-SB and Pl-SB from PreEx0, ^+^ *p* < 0.05 BAl-SB from Pl–Pl, *thick vertical bar* denotes exercise

There were significant increases in blood HCO_3_^−^ between baseline and the post-supplementation period in both trials involving NaHCO_3_ supplementation (Fig. 3, BAl-SB and Pl-SB) (average increase of 3.67 ± 0.46 mmol L^−1^; *p* ≤ 0.0001). The placebo group showed no changes. The blood HCO_3_^−^ in these groups was also significantly higher than that of the placebo condition (Pl–Pl) throughout both exercise bouts (*p* ≤ 0.01). No change was observed in blood pH between supplement groups or compared to baseline values prior to either exercise (Fig. 4). There was an increase in both blood HCO_3_^−^ concentration and blood pH at the end of the recovery period following BAl-SB and Pl-SB compared to baseline values and the Pl–Pl condition in both exercise bouts (*p* ≤ 0.01), with the exception of BAl-SB blood HCO_3_^−^ between baseline and 60 min following RSA (*p* = 0.07)). BAl-SB was the only treatment group to show significantly higher blood pH values (up to 0.07 ± 0.01 pH units; *p* ≤ 0.0001) throughout the first 10 min of recovery compared to Pl–Pl, in both exercise tests.

### Repeated sprint ability test (RSA)

No differences in peak power output (PPO) or average power output (APO) were observed between supplement groups during each of the 56-s sprints of the RSA test (Table 1). There were also no differences in the total work done between either or combined supplement groups with a comparison of total work done (TW) (BAl-Pl, 22,136.25 ± 1,757.01 J; BAl-SB, 22,487.00 ± 1,724.30 J; Pl–Pl, 23,586.00 ± 1,831.58 J; Pl-SB, 23,068.50 ± 1,749.20 J).

**Table 1:** Peak power output (PPO) and average power output (APO) (W) achieved for each of the 5 × 6 s sprints between supplement groups during the repeated sprint ability (RSA) test

	BAl-Pl	BAl-SB	Pl–Pl	Pl-SB
PPO (W)				
Sprint 1	987 ± 74.70	970 ± 71.32	1,028 ± 70.53	1,000 ± 72.20
Sprint 2	969 ± 75.43	976 ± 84.57	989 ± 73.16	1,018 ± 70.28
Sprint 3	948 ± 72.27	946 ± 78.04	966 ± 61.07	969 ± 58.40
Sprint 4	933 ± 72.72	927 ± 69.18	938 ± 58.96	911 ± 58.60
Sprint 5	884 ± 73.99	884 ± 60.74	913 ± 51.96	904 ± 64.19
APO (W)				
Sprint 1	803 ± 67.13	813 ± 64.23	873 ± 73.35	857 ± 64.13
Sprint 2	774 ± 61.74	792 ± 56.67	826 ± 69.25	826 ± 60.50
Sprint 3	732 ± 59.13	749 ± 59.51	773 ± 62.57	757 ± 61.34
Sprint 4	709 ± 54.24	705 ± 57.31	749 ± 57.35	721 ± 56.57
Sprint 5	672 ± 55.40	690 ± 53.18	710 ± 46.26	684 ± 56.45

Values expressed as mean ± SEM

### Cycling capacity test (CCT_110 %_)

**Fig. 5:**
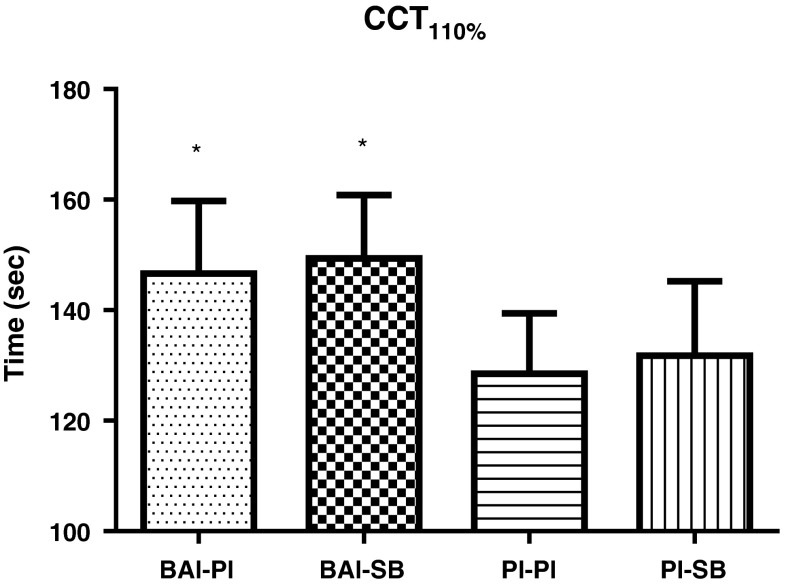
Time to exhaustion (TTE) (s) results for the different supplement groups during the cycling capacity test (CCT_110 %_). Values expressed as mean ± SEM. *p < 0.05 from Pl–Pl

A greater TTE by 18.13 ± 6.75 s (+14 %) (*p* = 0.005) was measured in the BAl-Pl compared to Pl–Pl (147 ± 13.16 and 129 ± 10.94, respectively) during the CCT_110 %_ (Fig. 5). Although the addition of NaHCO_3_ to β-alanine further increased TTE by 2.75 ± 4.61 s (+2 %) (*p* = 0.02 compared to Pl–Pl), there was no statistical difference between BAl-Pl and BAl-SB (*p* = 0.57). No change in TTE was determined in Pl-SB treated group (132 ± 13.48 s)(+2 %) compared to the Pl–Pl condition (*p* = 0.51).

### Plasma lactate

**Fig. 6:**
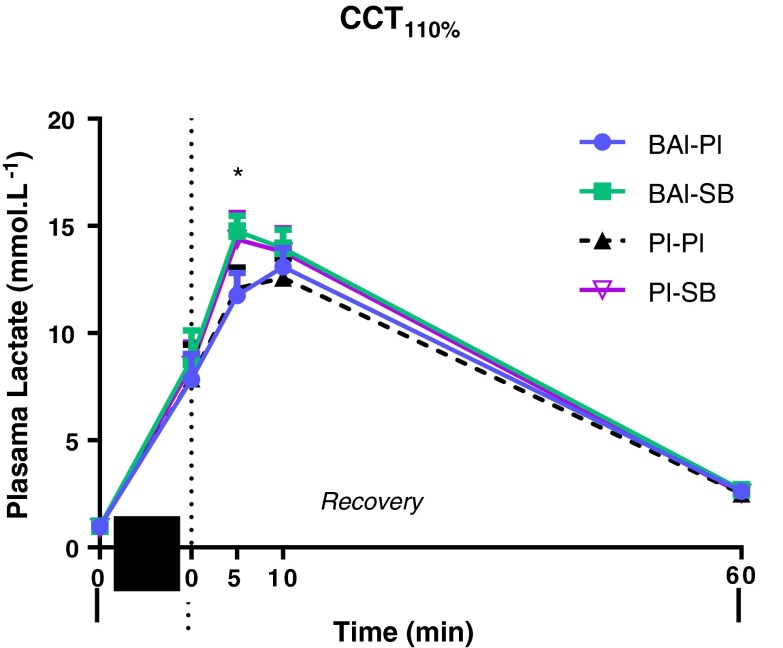
Plasma lactate concentration (mmol L^−1^) for the different supplemental groups during the cycling capacity test (CCT_110 %_). Values expressed as mean ± SEM. *p < 0.05 BAl-SB from Pl–Pl*. Thick vertical bar* denotes exercise

Plasma lactate concentrations (mmol L^−1^) were elevated in all treatment groups following the completion of exercise in both the RSA (data not shown) and CCT_110 %_ (Fig. 6), and remained significantly elevated compared to baseline at the end of the recovery period. A difference was observed at the 5th min of the recovery period following the CCT_110 %_ protocol, with BAl-SB higher than Pl–Pl by 2.66 ± 1.20 mmol L^−1^ (*p* = 0.04). Although the Pl-SB treated groups also showed an apparent increase in plasma lactate (+2.28 ± 1.61 mmol L^−1^) following this test, this was non-significant (*p* = 0.18).

## Discussion

This study demonstrated an increase in both intracellular and extracellular buffering potential following chronic β-alanine (6 weeks) and acute NaHCO_3_ (90 min) supplementation, respectively. Furthermore, the combined supplementation induced a similar buffering potential to that of the sum of the individual supplementation protocols. Improvements in exercise performance following the combined supplementation protocol, however, were equivocal and indicate that aggregating the supplementation protocol may not have a cumulative effect on physiochemical buffering capacity. The improvement in the CCT_110 %_ performance and not the RSA protocol may be a reflection of the modality of HIE which may influence metabolism differently and consequently have different impacts on intracellular and extracellular buffering systems. Furthermore, it could demonstrate that fatigue may not be pH related in some instances of HIE.

Muscle carnosine concentrations increased by 62 and 88 % in the gastrocnemius and soleus, respectively, following 6 weeks of supplementation with β-alanine. This is comparable to direct measures in vastus lateralis biopsies of untrained subjects (Harris et al. 2006; Hill et al. 2007) and indirect MRS gastrocnemius and soleus muscle of both trained (Derave et al. 2007) and untrained males (Baguet et al. 2009), following chronic supplementation of β-alanine. The increase in carnosine content in the current study was higher than previous reports using MRS technology, with Baguet et al. (2009) showing a 23 and 39 % increase, and Derave et al. (2007) a 37 and 47 % increase in the gastrocnemius and soleus muscles, respectively. This may be due to differences between β-alanine supplementation protocols where 6 weeks and a total dosage of 224 mg were utilised in this study compared to shorter duration and lower dosages implemented in previous studies.

The efficacy of the NaHCO_3_ supplementation employed in this study was demonstrated by a significant increase in blood HCO_3_^−^ following the 90-min pre-exercise supplementation period in both the BAl-SB and Pl-SB groups compared to placebo (Fig. 3). The elevation in blood HCO_3_^−^ in the current study (5.99 mmol L^−1^) following NaHCO_3_ ingestion was of a similar magnitude to the mean increase of 5.3 mmol L^−1^ reported in a meta-analysis of human NaHCO_3_ loading studies which also used a 300 mg kg^−1^ dose (Matson and Vu Tran 1993). Although other investigations have reported plasma pH increases of ~0.05 units following similar HCO_3_^−^ supplementation regimes, discrepancies in HIE performance have resulted in either positive (Bishop et al. 2004; Lindh et al. 2008; Siegler et al. 2010) or no change (Matsuura et al. 2007; Price et al. 2003; Sale et al. 2011). A lack of change in blood pH between pre-acute supplementation and pre-exercise was observed in this study, indicating that despite a significant rise in blood HCO_3_^−^, metabolic alkalosis was not being achieved (see Fig. 4).

Ingestion of NaHCO_3_ elevated blood pH by ~0.07 pH units 60 min following exercise in both HIE tests, along with a tendency to cause higher blood HCO_3_^−^ concentrations. This suggests that a longer time between acute supplementation and exercise commencing may have been more suitable in this study to induce an alkalotic state, which may in turn assist in improving HIE performance parameters. Extended ingestion protocols (>90 min) have been previously investigated, with metabolic alkalinity achieved prior to exercise; however, the performance enhancing abilities of NaHCO_3_ even with longer supplementation protocols employed remain equivocal (Gaitonos et al. 1991; Sale et al. 2011).

To our knowledge, this is the first study to examine the co-ingestion of β-alanine and NaHCO_3_ on intermittent HIE performance parameters. PPO and APO were not different between treatment groups during each of the 5 × 6 s sprints performed during the RSA test, or in terms of total work performed. Gaitonos et al. (1991) also reported that NaHCO_3_ supplementation did not induce performance benefits when using a similar exercise protocol (10 × 6 s sprints every 36 s) in male subjects. Although Gaitonos et al. (1991) assessed running performance, rather than cycling performance as used in this study; both used similar metabolic pathways in order for the effect of supplementation to be seen (Fitzsimons et al. 1993). This is contrary to an identical RSA exercise protocol employed by Bishop et al. (2004) demonstrating that the oral ingestion of NaHCO_3_ in active female hockey players increased PPO compared to the placebo condition during the last three of the 5 × 6 s sprints performed, indicating a potential specific conditioning of training-dependant response.

Sweeney et al. (2010) examined the effect of 5 weeks β-alanine supplementation using a similar total dose (224 vs. 238 g) on RSA in active males. Consistent with the present findings, β-alanine supplementation alone did not influence repeat sprint performance, with no change in power output observed. Co-ingestion with NaHCO_3_ in our study was unable to improve performance, demonstrating no influence of the treatment strategy on intermittent HIE.

The absence of a difference between treatments indicates either a potential gain in both intra- and extracellular buffing capacity, represented by observed elevations in muscle carnosine and blood bicarbonate, was either insufficient to bring about an increase in performance, or that decreases in pH is not the predominant limiting factor in intermittent HIE. Previous research observing high-intensity repeated sprint performance has also attributed fatigue to the availability and resynthesis of phosphocreatine (PCr), in addition to the effects of acidosis (Derave et al. 2010; Sweeney et al. 2010). During a single bout (6 s) of high-intensity exercise, the utilisation of PCr and anaerobic glycolysis each account for a majority of the energy source required (Sweeney et al. 2010). Repetition of short-duration sprints may result in a greater depletion of PCr than would occur during a single maximal effort (Derave et al. 2007). The rest between sprints during the RSA (24 s) may not have been long enough to resynthesis PCr, as this process typically takes minutes rather than seconds (Derave et al. 2007). Hence, it is possible that PCr depletion may have been a greater cause of fatigue than acidosis during the RSA protocol implemented (5 × 6 s interspersed with 24 s rest), and as a result, any potential ergogenic effects of β-alanine and NaHCO_3_ as physiochemical buffers during intermittent HIE may not have been detectable.

β-alanine supplementation significantly increased TTE during the CCT_110 %_ test with a 14 % improvement. This compares favourably with identical continuous HIE cycling protocols implemented by Sale et al. (2011) who also reported a 15 % increase following a 4-week supplementation period, and Hill et al. (2007) who showed a 13 % increased enhancement in TTE 4 weeks following supplementation, with a further 3 % increase after a 6-week supplement extension. Cycling capacity protocols, such as the one employed in these studies, can be considered beneficial as they induce a large accumulation of acidic by-products resulting in a drop in intracellular pH, such that the potentially ergogenic effects of β-alanine are influential.

In contrast to the effect of β-alanine on continuous HIE, NaHCO_3_ ingestion alone did not improve performance parameters relative to that of the Pl–Pl condition. Earlier studies show no effect of NaHCO_3_ on exercise performance using cycling protocols where participants cycle to exhaustion at both 110 % (Robergs et al. 2005; Sale et al. 2011) and 120 % *W*_max_ (Siegler et al. 2007) has previously been observed, despite the clear stimulus of increased H^+^ production in the working muscle cells. Consistent with the report of Sale et al. (2011), the results of this investigation support the ability to increase continuous HIE capacity following the co-ingestion of β-alanine and NaHCO_3_ above that of NaHCO_3_ alone, but not compared to β-alanine alone, with only a 2 % increase (~3 s) in TTE attained. Whilst this magnitude of elevation was not statistically significant (with 95 % confidence limits), it could be postulated that in terms of athletic performance small increases in TTE may still be important.

An increased buffering capacity may improve metabolic conditions for sustained elevated glycolytic energy production during the final stages of maximal effort exercise, and result in elevation of lactate ions in the active muscles and blood (Price and Simons 2010; Siegler et al. 2010; Van Thienen et al. 2009). Immediately following both the RSA test and CCT_110 %_, plasma lactate values were not different between groups. However, 5 min following the CCT_110 %_ (Fig. 6), a marked increase in plasma lactate was observed in NaHCO_3_ supplemented groups compared with Pl–Pl, significant only in the BAl-SB treatment.

The lack of any observed change in plasma lactate concentration between the BAl-Pl and Pl–Pl groups following both the RSA and CCT_110 %_ exercise protocols, suggests that enhanced intracellular buffering capacity did not promote increased glycolytic metabolism. Others have also reported no change in plasma lactate following HIE post chronic supplementation with β-alanine, although they did report performance benefits (Sweeney et al. 2010). Improvements in TTE in the current studies CCT_110 %_ test may therefore indicate a potential mechanism of action other than enhanced intracellular buffering capacity in which β-alanine may provide ergogenic effects during continuous HIE.

In summary, there was no aggregating effect of the combined supplementation protocol on performance or metabolism, although the ingestion of β-alanine and NaHCO_3_ elevated both intra- and extracellular buffering capacity potential, by increasing concentrations of muscle carnosine and blood HCO_3_^−^, respectively. Alterations in buffering potential did coincide with performance enhancements in continuous fatiguing exercise, with TTE improved following β-alanine supplementation. No changes were found in performance parameters between the treatment combinations during maximal intermittent sprint exercise. No change in blood pH following NaHCO_3_ ingestion in the BAl-SB and Pl-SB groups demonstrates that an alkalotic state was not produced prior to exercise commencing, suggesting that the time between acute feeding and exercise implemented in this study may not be long enough to alter extracellular alkalinity, which in turn may have limited the potential for performance improvements. This may indicate that pH may not be a mechanism that influences fatigue in all HIE protocols. Further investigations into the potential mechanisms in which β-alanine provide ergogenic effect during continuous HIE performance, including the role of muscle carnosine as an antioxidant, are warranted as other intramuscular influences such as alterations in ROS (by-products of antioxidant effects) may play an influential role in potentially enhancing exercise performance.

### **Author Contributions**

 JD; Experimental design, conducted experimental trials, data collection and analysis, manuscript preparation. TG; Conducted experimental trials, data collection. RMW; data analysis. CGS; Experimental design, conducted experimental trials, data collection, manuscript preparation.

## Abbreviations

ANOVA: Analysis of variance
APO: Average power output
BAl-Pl: β-Alanine + calcium carbonate
BAl-SB: β-Alanine + sodium bicarbonate
CCT_110 %_: Cycling capacity test
HIE: High-intensity exercise
MRS: Magnetic resonance spectroscopy
Pl-Pl: Calcium carbonate + calcium carbonate
Pl-SB: Calcium carbonate + sodium bicarbonate
PPO: Peak power output
RSA: Repeated sprint ability
TTE: Time to exhaustion
VO_2peak_: Peak oxygen consumption
